# Key drivers structuring rotifer communities in ponds: insights into an agricultural landscape

**DOI:** 10.1093/plankt/fbab033

**Published:** 2021-05-06

**Authors:** Gabriela Onandia, Sebastian Maassen, Camille L Musseau, Stella A Berger, Carla Olmo, Jonathan M Jeschke, Gunnar Lischeid

**Affiliations:** Research Platform Data Analysis and Simulation, Leibniz Centre for Agricultural Landscape Research (ZALF), Eberswalder Straße 84, Müncheberg 15374, Germany; Berlin-Brandenburg Institute of Advanced Biodiversity Research (BBIB), Königin-Luise-Straße 2-4, Gartenhaus, Berlin 14195, Germany; Research Platform Data Analysis and Simulation, Leibniz Centre for Agricultural Landscape Research (ZALF), Eberswalder Straße 84, Müncheberg 15374, Germany; Berlin-Brandenburg Institute of Advanced Biodiversity Research (BBIB), Königin-Luise-Straße 2-4, Gartenhaus, Berlin 14195, Germany; Berlin-Brandenburg Institute of Advanced Biodiversity Research (BBIB), Königin-Luise-Straße 2-4, Gartenhaus, Berlin 14195, Germany; Institute of Biology, Freie Universität Berlin, Königin-Luise-Straße 1-3, Berlin 14195, Germany; Department of Ecosystem Research, Leibniz Institute of Freshwater Ecology and Inland Fisheries (IGB), Müggelseedamm 310, Berlin 12587, Germany; Berlin-Brandenburg Institute of Advanced Biodiversity Research (BBIB), Königin-Luise-Straße 2-4, Gartenhaus, Berlin 14195, Germany; Department of Experimental Limnology, Leibniz Institute of Freshwater Ecology and Inland Fisheries (IGB), Zur alten Fischerhütte 2, Stechlin 16775, Germany; GEMA Center for Genomics, Ecology and Environment, Facultad de Ciencias, Universidad Mayor, Camino La Pirámide 5780, Santiago 8580745, Chile; Berlin-Brandenburg Institute of Advanced Biodiversity Research (BBIB), Königin-Luise-Straße 2-4, Gartenhaus, Berlin 14195, Germany; Institute of Biology, Freie Universität Berlin, Königin-Luise-Straße 1-3, Berlin 14195, Germany; Department of Ecosystem Research, Leibniz Institute of Freshwater Ecology and Inland Fisheries (IGB), Müggelseedamm 310, Berlin 12587, Germany; Research Platform Data Analysis and Simulation, Leibniz Centre for Agricultural Landscape Research (ZALF), Eberswalder Straße 84, Müncheberg 15374, Germany; Berlin-Brandenburg Institute of Advanced Biodiversity Research (BBIB), Königin-Luise-Straße 2-4, Gartenhaus, Berlin 14195, Germany; Institute for Environmental Sciences and Geography, University of Potsdam, Karl-Liebknecht-Straße 24-25, Potsdam 14476, Germany

**Keywords:** biodiversity, eutrophication, freshwater, water quality, zooplankton

## Abstract

Understanding the influence of environmental and spatial factors on the structure of aquatic communities remains a major challenge in community ecology. This study aims to identify main drivers of rotifer abundance and diversity in ponds embedded in an intensive agricultural landscape in Northeast Germany. We studied 42 ponds of glacial origin (kettle holes) covering a wide range of environmental parameters. The predominant factors structuring the rotifer metacommunity shifted from abiotic environmental filtering in spring to unstudied factors in autumn, while spatial factors remained less important. Fertilizer-driven salinization, internal nutrient recycling, primary productivity and sediment phosphorus release were the prevalent biogeochemical processes in the ponds. Both fertilizer-driven salinization and primary productivity negatively affected rotifer alpha diversity, and positively affected beta diversity. This impact was lower in forest ponds than in those surrounded by arable fields or grassland. However, rotifer diversity did not significantly differ among land-use categories. Our results indicate that the long-term impact of intensive agriculture in the region and the associated widespread eutrophication overrides the direct influence of land use on rotifer diversity but point to an indirect effect via fertilizer-driven salinization. Furthermore, this study highlights the role of ponds in enhancing regional biodiversity in agricultural landscapes.

## INTRODUCTION

Small lentic water bodies (<1 ha) are the most abundant type of inland waters but have been generally ascribed a minor role in the context of global nutrient cycling and biodiversity conservation (De Meester *et al.*, 2005; Downing *et al*., 2006, 2010). However, ponds have been increasingly recognized as hotspots for carbon and nutrient turnover in the landscape (Downing, 2010; Catalán *et al*., 2014; Premke *et al*., 2016; Obrador *et al*., 2018). Likewise, in the last decades, several studies have shown the importance of these habitats for biodiversity maintenance at the regional level (Oertli *et al*., 2002; Williams *et al*., 2004; Davies *et al*., 2008). Ponds are known to be sensitive to environmental changes (Angeler *et al*., 2010) such as salinity, temperature or macrophyte habitat structure (Duggan *et al*., 2001; Guisande *et al*., 2008; Kaya *et al*., 2010; Malekzadeh-Viayeh and Špoljar, 2012). They are also very vulnerable towards agricultural intensification and the associated fertilization and fragmentation of natural habitats, resulting in a loss of biodiversity (Scheffer *et al*., 2006; Céréghino *et al*., 2008; Stoate *et al*., 2009). A clear impact of land use on the species composition and abundance of different groups of aquatic organisms has been described in a variety of ecosystems. At the metacommunity level, for instance, beta diversity has been found to decline in aquatic communities subjected to eutrophication (Passy and Blanchet, 2007; Donohue *et al*., 2009). A possible explanation is that increased environmental harshness fosters the dominance of deterministic community assembly processes (niche filtering) over stochastic ones (ecological drift, dispersal or colonization/extinction dynamics) (Chase, 2007; Cook *et al*., 2018). While many studies focused on the effects of land use on the diversity of larger organisms like macroinvertebrates (Gołdyn *et al*., 2012; Hill *et al*., 2016) or macrophytes (Pätzig *et al*., 2012; Waldon, 2012; Altenfelder *et al*., 2014) in ponds, smaller invertebrates like zooplankton, and particularly rotifers, have been less frequently studied (Sahuquillo and Miracle, 2019). Rotifers are important constituents of aquatic food webs due to their high reproducibility and large abundance, but also because they link bacteria, flagellates and phytoplankton to secondary consumers (Conde-Porcuna *et al*., 2004; Sahuquillo and Miracle, 2019). Moreover, the rapid response of small zooplankton to environmental changes makes them valuable ecological indicators (Jeppesen *et al*., 2011).

Understanding the role of environmental and spatial factors in determining the structure of aquatic communities remains a major challenge in community ecology (Cottenie *et al*., 2003; Cardoso *et al*., 2017; Thompson *et al*., 2020). Local biotic (e.g. resource competition and predation; Shurin and Allen, 2001) and abiotic factors such as hydroperiod or salinity gradients (Frisch *et al*., 2006; Waterkeyn *et al*., 2008) as well as spatial factors related to mechanisms and processes at the regional scale e.g. dispersal (Hubbell, 2001), are important drivers of metacommunity structure. In addition, the dispersal ability of the community influences beta diversity and the assembly of aquatic communities in ponds (Leibold *et al*., 2004). Despite the recent progress made in metacommunity research, Logue *et al*. (2011) have stressed the need for extending empirical studies to underrepresented systems like temporary natural systems.

The young moraine landscape of Northeast Germany, dotted with thousands of kettle holes, provides an excellent model system to assess the relative importance of environmental versus spatial factors in determining the structure of the rotifer metacommunity. Kettle holes are small (<1 ha) shallow ponds scattered at very high density in the glacially formed landscapes of northern Europe, Asia and North America (Gala and Young, 2015; Mitsch and Gosselink, 2015). Up to 300 000 kettle holes (hereafter termed “ponds”) with a density of up to 40 per hectare have been described in Northeast Germany, a region where intensive agriculture is the predominant land use (Kalettka *et al*., 2001; Kalettka and Rudat, 2006). They exhibit important variations with respect to hydrogeomorphology, hydroperiod (ranging from episodic to permanent) and water quality (Kalettka and Rudat, 2006; Lischeid and Kalettka, 2012). These small water bodies are important aquatic-terrestrial interfaces for biogeochemical processes (McClain *et al*., 2003), hotspots for nutrient turnover (Reverey *et al*., 2016; Onandia *et al*. 2018) and for biodiversity (Gołdyn *et al*., 2012; Pätzig *et al*., 2012; Lozada-Gobillard *et al*., 2019).

The aims of this study were to: (i) quantify the relative importance of environmental versus spatial factors in determining the structure of the rotifer metacommunity, (ii) identify the biogeochemical processes prevalent in the water column of the ponds and their potential controlling factors (e.g. habitat structure, hydrogeomorphic factors, land use and seasonality) and (iii) assess the influence of the identified biogeochemical processes and their controlling factors on rotifer abundance, alpha and beta diversity. First, we hypothesize that due to the relatively small size of our study area and the presence of dispersal vectors, dispersal is not limited, and spatial factors, therefore, explain a minor proportion of rotifer metacommunity structure. Second, based on the prevalence of agricultural land use in our study area, we expect eutrophication-related processes, such as increased nutrient availability due to fertilizer application and the associated higher primary productivity, to prevail in the water column of the ponds and to be mainly controlled by land use in the vicinity of the ponds, being more prominent in those surrounded by arable fields. Third, given the described adverse effects of agricultural land use on aquatic organisms (Carpenter *et al*., 1998; Dodson *et al*., 2005; McGoff *et al*., 2013; Zhang *et al*., 2020), we hypothesize that eutrophication-related processes negatively affect rotifer abundance, alpha and beta diversity. Our study provides empirical insights into the factors structuring rotifer communities in ponds of varying hydroperiod, and thus into a group of small invertebrates and a type of natural system that are both underrepresented in metacommunity research.

## METHOD

### Study site and data collection

**Fig. 1:**
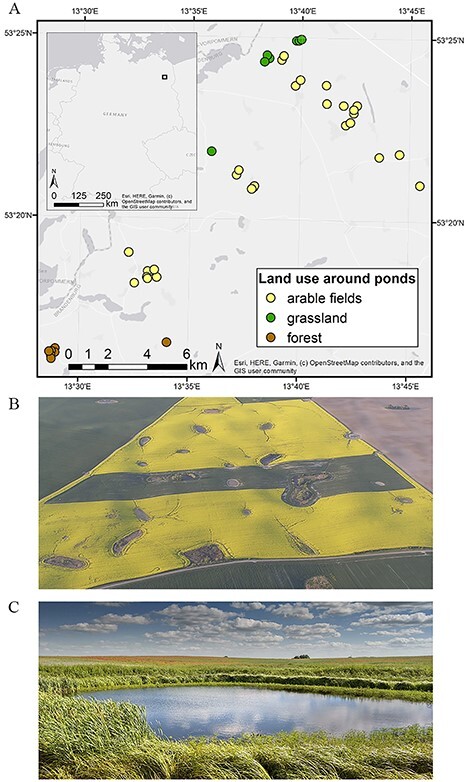
(**A**) Geographical location of the studied ponds in Uckermark (Germany). Colors correspond to the three land use categories surrounding the ponds: arable fields (*n* = 29), grassland (*n* = 6) and forest (*n* = 7). (**B**) Aerial view of part of our study area (photo by G. Verch) and (**C**) kettle hole (photo by G. Onandia).

The study was performed in the AgroScapeLab Quillow (ASQL), i.e. at the catchment area of the Quillow stream in the Uckermark, Northeast Brandenburg, Germany (Fig. 1). The predominant land use in the area is intensive agriculture (75%), mostly arable fields, with only small patches of forest and grassland (Merz and Steidl, 2015). The soil of this area is mainly loamy and partly sandy. As a model system for this study, we selected 42 fishless ponds within an area of 220 km^2^ belonging to the ASQL, an experimental area established by the Berlin-Brandenburg Institute of Advanced Biodiversity Research (BBIB) with a network of sites for the investigation of biodiversity and ecosystem functioning in agricultural environments (ScapeLabs Experimental Platform, 2020). The selected ponds varied with respect to habitat structure (e.g. habitat complexity and canopy cover over the ponds) and a number of hydrogeomorphic characteristics (e.g. hydroperiod, water level, hydrogeomorphic type, shore width, shore slope and maximum depth of the pond basin) as well as land use in their vicinity. Ponds typically fill up during spring as a result of groundwater inflow, snowmelt as well as runoff on frozen soil, and lose water by evapotranspiration and regional groundwater discharge, drying up during the hydrological summer under negative climatic water balance (Kalettka and Rudat, 2006). It should be noted that both flooding and drying dates vary greatly between years, leading to unpredictable dry–wet cycles (Reverey *et al*., 2016). As a consequence, the ponds in this region are typically fishless.

The ponds were sampled twice, in late spring 2017 between May 31st and June 21st, and in early autumn 2017 between October 3rd and 12th. Due to the temporary nature of most of the ponds and logistical constraints (seven ponds located in the forest could only be accessed in spring), the sample size varied across sampling periods. Out of the 42 selected ponds, 27 ponds were sampled in both spring and autumn, 9 were only sampled in spring and 6 were only sampled in autumn. The mean annual accumulated precipitation in 2017 was 459 mm, slightly less than the average annual accumulated precipitation between 1981 and 2010, which was 514 mm (Station Angermünde, Uckermark, DWD 2020). In both sampling periods, we measured a set of physical–chemical, habitat structure and hydrogeomorphic variables in the ponds and determined land use in their vicinity. Among physical–chemical parameters, pH, electric conductivity (EC), water temperature and dissolved oxygen concentration (DO) were measured *in situ* using electronic meters (SenTix 81 pH electrode coupled to a ProfiLine pH 3 110, Cond 3 110 sensor coupled to a TetraCon 325 and CellOx 325 sensor coupled to a ProfiLine Oxi 3 205 portable meter, respectively (Xylem Analytics Germany Sales GmbH, WTW, Weilheim, Germany)). Alkalinity was also determined on site using a field test set (MSD Sharp & Dohme GmbH, Haar, Germany). In addition, 16 parameters were analyzed *ex situ* from water samples of each pond in accordance with German standard methods (DIN ISO standards). Soluble reactive phosphorus (SRP), ammonium (NH_4_-N) and spectral absorption coefficient (SAC) were measured spectrophotometrically using a SPECORD 210 plus (Analytik Jena AG, Germany). Additionally, sulfate (SO_4_), nitrate (NO_3_-N), bromide (Br) and chloride (Cl) were analyzed by ion chromatography with an 882 Compact IC plus (Deutsche Metrohm GmbH & Co. KG, Filderstadt, Germany). Total Fe, Na, K, Mg and Ca were analyzed using ICP-OES (ICP-iCAP 6 300 DUO, Thermo Fisher Scientific GmbH, Schwerte, Germany). Total phosphorus (TP) was analyzed as soluble phosphorus after microwave digestion (Gallery Plus, Microgenics GmbH, Thermo Fisher Scientific GmbH, Schwerte, Germany). Dissolved organic carbon (DOC), total organic carbon (TOC) and total nitrogen (TN) were measured using elemental analysis with chemiluminescence detection (TOC-Vcph, Shimadzu Deutschland GmbH, Duisburg, Germany). Water samples for chlorophyll-*a* (Chl-*a*) and pheophytin (Pheo) determination were prefiltered through a 100-μm mesh to remove larger detritus and biota. Pigment concentrations were determined from water samples filtered onto glass-fiber filters (GF/F, Cytiva Europe GmbH, Freiburg im Breisgau, Germany), that were immediately placed inside a glass vessel and stored at −80°C in the dark until they were processed. Chl-*a* and Pheo were extracted with 96% ethanol and measured spectrophotometrically (DIN 38 412–16, 1985).

With regard to habitat structure variables, habitat complexity was estimated as the number of macrophytes functional groups (submerged macrophytes, free-floating macrophytes and helophytes) present at the inundated area of the ponds, that is, 0–3. Common helophytes observed during the study were *Phragmites australis* (Cav.) Trin ex Steudel, *Phalaris arundinacea* L., *Typha* sp, *Rorippa amphibia* (L.) Besser, *Oenanthe aquatica* (L.) Poiret and *Carex* spp. The dominant submerged macrophytes were *Ceratophyllum submersum* L., *Ceratophyllum demersum* L. and *Potamogeton* spp. The free-floating macrophytes *Lemna minor* L., *Lemna trisulca* L., *Spirodela polyrhiza* (L.) Schleid were also frequently observed. Canopy cover over the ponds was measured only in spring using a spherical crown concave densiometer (Concave Model C, Forestry Suppliers, Inc., Jackson, Mississippi, USA). Canopy cover was quantified by counting the number of “canopy” dots on a grid lying on a concave mirror reflecting the canopy. For each pond, the canopy cover was measured in three different locations, with four canopy readings facing each cardinal direction. The measures were averaged at the location level and then at the site level. For the purpose of the study, values of canopy cover were divided into four quartile classes.

Regarding hydrogeomorphic variables, we classified the ponds according to their hydroperiod in the four following categories from Kalettka and Rudat (2006): episodic (long drying-up period starting in early summer, except in very wet years), periodic (short drying up starting in autumn, except in very wet years), semi-permanent (drying up only every few years following a perennial deficit in precipitation) and permanent (no drying up). In addition, water level was visually estimated during our two sampling periods as the percentage of inundation of the pond basin, using the following six categories: 0%, <20%, 20–<50%, 50–<100%, 100% and >100%. Ponds were also classified based on their hydrogeomorphic characteristics (hydrogeomorphic type, shore width, shore slope and maximum depth of the pond basin) following the methodology established by Kalettka and Rudat (2006). Pond area was calculated by measuring the maximum length and width or the circumference at the top of the shoreline according to Kalettka and Rudat (2006), depending on whether the pond area was closer to a rectangle or disc, respectively.

Furthermore, we classified the land use in the catchment of the ponds, using three categories: arable fields, grassland and forest. The categories were determined based on the InVeKoS data set, i.e. anonymized GIS data (field sketches), provided by the Ministry for Infrastructure and Agriculture of the Federal State of Brandenburg, and verified on site. Crops at the arable fields in the year of the study (2017) were wheat, corn, rape and barley. Forest areas comprised mixed deciduous and coniferous tree species.

Integrated zooplankton samples were taken by collecting water at different locations in order to account for microhabitat heterogeneity within ponds. Zooplankton samples were concentrated by filtering 2.9–10 L of water through a 30-μm nytal mesh and preserved in 4% formalin. Since most rotifer species can be found within the 30–280-μm size range, samples were filtered prior to their analysis through a 280-μm nytal mesh to exclude mesozooplankton (mainly crustaceans). At least three *ca.* 0.8 mL concentrated aliquots per sample were analyzed using the image-based flow cytometer FlowCAM^®^ VS Portable (Fluid Imaging Technologies, Inc., Maine, USA), equipped with a 4× objective and a 300-μm flow cell, and run for 5 minutes each in auto-image mode. Obtained images were processed using VisualSpreadsheet^®^ Particle Analysis Software v2.4.1. Images of debris, phytoplankton, copepod nauplii as well as artifacts and repeated images of rotifers were removed. To ensure precise taxonomic identification, rotifers were identified manually to the genus level and, when possible, to the species level following Koste (1978). Rotifers belonging to the order Bdelloidea were considered as a single taxon. The volume analyzed and the total abundance of rotifers in the samples were calculated by combining results of the analysis software and the sampling concentration procedure, while the abundance of individuals belonging to the different genera was calculated based on manually sorted images. The average counts of each aliquot (*n* ≥ 3) were used to calculate the abundance of the different rotifer genera as well as total rotifer abundance (thereafter rotifer abundance; individuals L^−1^) for each sampling site and date.

### Data analysis

The statistical analyses described in this section were performed using R software version 3.6.1 (R Development Core Team, 2019). Since some organisms could not be identified to the species level, individuals were grouped at the genus level for estimating rotifer abundance and diversity and all subsequent analyses. We calculated two measures of alpha diversity: (i) rotifer richness was estimated as the total number of rotifer genera found in each pond at each sampling period; and (ii) the Shannon index was calculated based on rotifer genus abundance. The Whittaker index was used to quantify beta diversity, being the average of a matrix of pairwise dissimilarities for each pond. Shannon and Whittaker indices were computed with the package “vegan” (Oksanen *et al*., 2019). Generalized linear mixed-effects models (GLMMs; R package “MASS”; Venables and Ripley, 2002) were used to test significant effects in all described analyses. The distribution of the response variables was explored, and the link functions were chosen accordingly for these and all subsequently described GLMMs (Zuur *et al*., 2009). Namely, we used a Gaussian distribution (link function *identity*) in the models in which the response variable was a physical–chemical parameter, beta diversity or the Shannon index. A quasi-Poisson distribution (link function *log*) was used in the models in which the response variable was rotifer richness or abundance to deal with overdispersion.

### Role of environmental versus spatial factors structuring the rotifer metacommunity

To calculate the extent of dissimilarity and the relative contribution of taxa to the divergence between communities among sampling periods (e.g. seasons), we performed an analysis of similarity (ANOSIM) and a analysis of similarity percentages (SIMPER). To that end, we calculated a similarity matrix using Bray–Curtis similarity coefficients based on rotifer abundance. Next, to disentangle the role of environmental *versus* spatial factors on the structure of the rotifer metacommunity of ponds, we carried out one variation partitioning for each sampling period (Peres-Neto *et al*., 2006). First, spatial variables were obtained by transforming latitude and longitude data into Moran’s Eigenvector Maps (MEMs), which consist of a matrix of positively autocorrelated orthogonal variables of different spatial scales (Dray *et al*., 2006). While MEMs with high eigenvalues represent broad-scale patterns among sampling points, MEMs with small eigenvalues represent fine-scale patterns (Griffith and Peres-Neto, 2006). Eigenvalues are obtained sequentially for the spatial arrangement defined by the abovementioned matrix as the set of values with the largest Moran coefficient achievable by any set that is uncorrelated with the preceding eigenvectors (Griffith and Peres-Neto, 2006). The highest obtained eigenvalue has, therefore, the largest Moran coefficient, while the smallest obtained eigenvalue has the largest negative Moran coefficient. MEMs, therefore, represent spatial structures that can be related to processes such as dispersal (Dray *et al*., 2006) and can be directly used as spatial predictor variables in regression or canonical models to depict spatial scale variation in metacommunity structure. Second, for each sampling period, three matrices were built: (i) a spatial matrix with MEMs; (ii) an environmental matrix, with all the environmental variables (except SAC and canopy cover), in which the land use, depth and vegetation type were dummy variables and the rest were continuous; and (iii) a relative abundance rotifer matrix. Abundance data were Hellinger-transformed and when necessary, environmental data were also transformed using logarithms or the arcsine of the square root. As a third and last step, we performed distance-based redundancy analysis (dbRDA) to explain the variation of each relative abundance rotifer matrix in relation to the environment and space. At this step, variables from environmental and spatial data matrices went through a forward selection process prior to each variation-partitioning analysis. Variation partitioning allows quantifying the percentage explained purely by the environmental component, purely by the spatial component, by the overlap between these two components, as well as the unexplained variation (residuals). These analyses were performed using the R packages “vegan,” “ade4” (Bougeard and Dray, 2018) and “adespatial” (Dray *et al*., 2019).

### Identifying biogeochemical processes and their controlling factors

To identify prevalent biogeochemical processes in the ponds, we performed an Isomap analysis (Isometric Feature Mapping) with the physical–chemical data using the R package “vegan.” Unlike other dimensionality reduction methods frequently used in ecology (e.g. principal component analysis, classical/nonmetric multidimensional scaling or self-organized maps), Isomap analysis can adequately tackle nonlinear relationships without a priori assumptions on the intrinsic data dimensionality (Mahecha *et al*., 2007). According to the decision tree proposed by these authors when performing multidimensional scaling, Isomap analysis represents the most suitable option for analyzing our data set given that: (i) an unsupervised approach was followed, i.e. no additional information was used; (ii) we opted for the geodesics approach and (iii) using the component scores as a quantitative measure for the respective effect size requires preservation of the input metric. Further information on this nonlinear ordination method based on the classical multidimensional scaling approach is provided by Tenenbaum *et al*. (2000), Mahecha *et al*. (2007) as well as Lischeid and Kalettka (2012). With Isomap, Euclidean distances to the next *k* data points are determined for every single instance. A geodesic distance matrix is set up that analyzes the nonlinear structure of that manifold with a piece-wise linear approach. A trial-and-error approach is then used to determine the optimal *k* value, which provides the optimal performance of the nonlinear projection. In our study, the optimal *k* value used for the Isomap analysis was 54. The Isomap components can be interpreted as quantitative measures of the effects of single processes corresponding to linear principal components. These interpretations are based on loadings, i.e. slope coefficients of the relationships between physical–chemical parameters and component scores. The loadings were obtained using GLMMs that were built for each Isomap component and each physical–chemical parameter, including the pond name and its geographical coordinates as random effects. Prior to analysis, all data were z-transformed and the few missing values replaced by zero since the mean of z-transformed data is always zero. The physical–chemical parameters having significant effects (estimates) on Isomap components were used for interpreting the biogeochemical process that each component explained (significance threshold was Bonferroni-corrected). Thus, we aimed at using the components for identification of the prevailing processes, and at using the component scores as quantitative measures of the respective effect size. The criterion to retain a certain number of components was to maximize the explained variance while selecting components considered potentially relevant for the rotifer community based on our hypotheses, such as those processes associated with agricultural land use and eutrophication.

Next, we tested the effects of potential controlling factors on the prevalent biogeochemical processes in the ponds. To assess land use effects, only the spring sampling period was considered, as forest ponds could not be sampled in autumn. For the same reason, only ponds in arable fields and grasslands were considered to assess the effects of habitat structure, hydrogeomorphic factors and seasonality. Potential controlling factors were included as fixed factors in the models, while the pond name and/or its geographical coordinates were included as random effects. The Isomap derived results were cross-validated by testing the effects of significant controlling factors (i.e. factors significantly affecting Isomap components) on water physical–chemical parameters. The pond name and/or its geographical coordinates were included as random effects.

### Effects of biogeochemical processes and their controlling factors on rotifer communities

We tested the effects of the four main Isomap components (used as proxies of biogeochemical processes, see above) on rotifer abundance, richness, Shannon index and Whittaker index. Forest ponds were excluded from this analysis, as they were only sampled in autumn. For each estimate, the four Isomap components were used as fixed effects and the pond name and its geographical coordinates as random effects. We also tested the effects of significant controlling factors on rotifer abundance and diversity estimates. To assess land use effects, only the spring sampling period was considered, as forest ponds could not be sampled in autumn. For the same reason, only ponds in arable fields and grasslands were considered to assess the effects of habitat structure, hydrogeomorphic factors and seasonality. The pond name and/or its geographical coordinates were included as random effects.

## RESULTS

### Environmental variables and rotifer communities

Physical–chemical parameters varied substantially across the studied ponds, often showing a skewed distribution with outlying maximal values (Table I). For instance, phosphorus concentrations (SRP and TP) were relatively high, whereas median values of nitrogen species (NO_3_-N, NH_4_-N, TN) were relatively low, with some outliers. Important variations were also found with regard to habitat structure and hydrogeomorphic characteristics (Fig. S1).

**Table I:** Mean and standard deviation of physical–chemical parameters along with rotifer abundance and diversity indices in the studied ponds in spring (*n* = 36) and autumn (*n* = 33)

Parameter	Spring Mean ± SD	Autumn Mean ± SD
**Environmental parameters**		
pH	7.0 ± 0.9	7.0 ± 0.7
EC (μS cm^−1^)	495.0 ± 296.3	541.9 ± 278.9
DO (mM)	3.8 ± 2.4	5.1 ± 3.4
Water temperature (°C)	18.9 ± 3.1	11.3 ± 1.2
Cl (mM)	23.5 ± 16.6	23.0 ± 12.2
TN (mM)	3.0 ± 1.7	3.1 ± 2.3
NO_3_-N (mM)	0.1 ± 0.5	1.0 ± 2.5
NH_4_-N (mM)	0.5 ± 1.0	0.3 ± 0.7
TP (mM)	0.9 ± 0.6	0.8 ± 0.5
SRP (mM)	0.7 ± 0.7	0.7 ± 0.6
SO_4_ (mM)	55.9 ± 69.7	47.9 ± 65.4
Na (mM)	8.3 ± 6.1	10.7 ± 5.5
K (mM)	10.1 ± 6.9	18.5 ± 10.3
Mg (mM)	6.7 ± 5.0	10.4 ± 5.5
Ca (mM)	55.7 ± 44.4	82.6 ± 50.8
TFe (mM)	0.4 ± 0.6	1.3 ± 1.3
DOC (mM)	35.7 ± 16.2	24.5 ± 10.3
TOC (mM)	37.4 ± 18.1	26.0 ± 11.1
SAC (mM)	116.3 ± 51.2	82.3 ± 39
Chl-*a* (μM)	10.8 ± 22.6	32.8 ± 54.1
Pheo (μM)	12.0 ± 26.1	14.5 ± 17.5
Alkalinity (mM)	5.3 ± 4.3	5.0 ± 2.7
Total hardness (mM)	3.3 ± 2.6	5.0 ± 2.9
**Rotifer abundance and diversity**		
Rotifer abundance (ind L^−1^)	343.5 ± 728.2	328.1 ± 867.5
Rotifer richness	4.7 ± 3.1	2.9 ± 2.4
Shannon index	1.0 ± 0.6	0.6 ± 0.6
Whittaker index	0.7 ± 0.1	0.8 ± 0.1

**Fig. 2:**
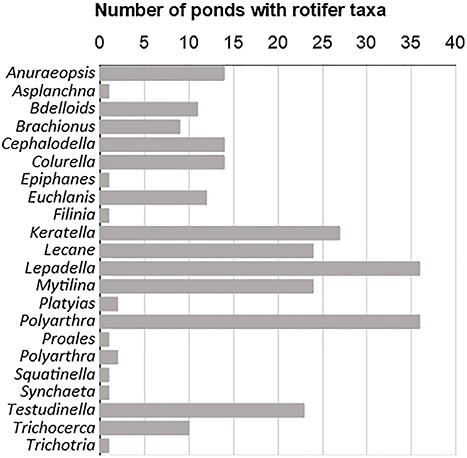
Presence of rotifer taxa (frequency) throughout the study period in the studied ponds.

A total of 21 monogonont rotifer genera belonging to 15 families were identified in the 42 ponds during the study period (Fig. 2 and Fig. S2). Examples of identified taxa using FlowCAM^®^ technology are given in Fig. S3. No taxon was ubiquitous across all ponds, although some genera showed high incidences, such as *Polyarthra*, *Lepadella* and *Keratella*, which were found in 28, 27 and 26 sites, respectively, across all land use and hydroperiod categories as well as in both sampling periods (Fig. 2 and Fig. S2). On the other hand, the genera *Asplanchna*, *Epiphanes*, *Filinia*, *Proales*, *Synchaeta*, *Squatinella* and *Trichotria* were recorded in one single pond and thus restricted to a specific land use and season (Fig. S2). Approximately, half of the genera were represented by single species: *Anuraeopsis* (i.e. *A. fissa* Gosse), *Ephiphanes* sp., *Filinia* (*F. longiseta* Ehrenberg), *Platyias* (i.e. *P. quadricornis* Ehrenberg), *Proales* sp., *Ptygura* sp., *Squatinella* (*S. cf rostrum* Schmarda), *Synchaeta* sp., *Testudinella* (*T. patina* Hermann) and *Trichotria* (*T. tetractis* Ehrenberg). Rotifer abundance varied substantially across ponds but was generally low (Table I, Fig. S2). Mean abundance during the study period was highest for *Keratella*, followed by *Polyarthra*, *Lecane*, *Lepadella* and bdelloids in descending order (Fig. S2).

### Role of environmental versus spatial factors structuring the rotifer metacommunity

Significant differences were found among the communities of spring and autumn (ANOSIM *R* = 0.081, *P* < 0.005), and the SIMPER showed an overall average dissimilarity of 80.7%. Only four genera contributed more than 50% to this dissimilarity: *Polyarthra*, *Keratella, Lepadella* and *Lecane*, which were abundant and very common in our ponds. In spring, *Keratella* was the genus that most frequently dominated the rotifer assemblage, accounting for at least 50% of rotifer abundance in one-third of the ponds. In contrast, *Polyarthra* made up for 50% of rotifer abundance in 38% of the ponds in autumn (Fig. S2).

**Fig. 3:**
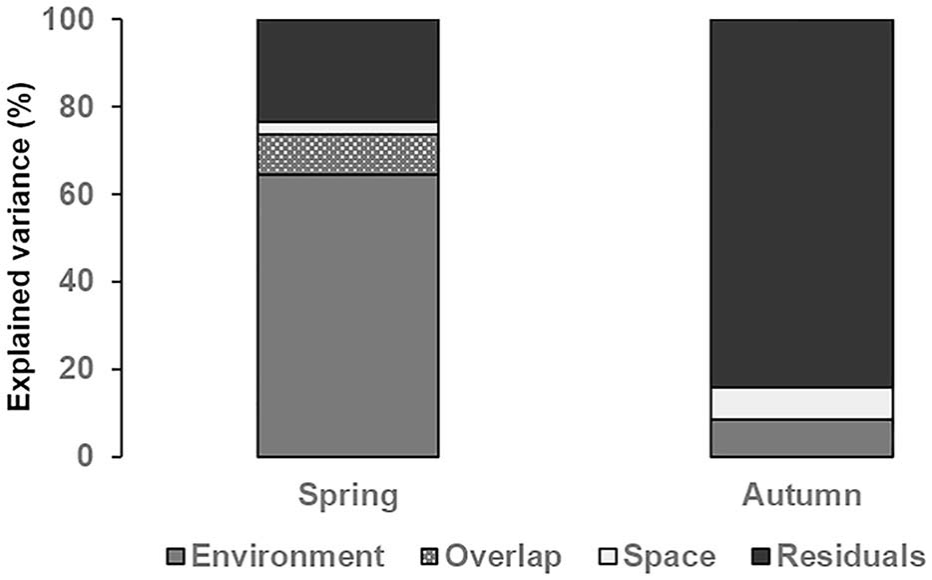
Results of variation partitioning (%) analyses of rotifers for each sampling season explained by: pure environmental variation (Environment), pure spatial variation (Space), an overlap between these components (Overlap), and the unexplained variation (Residuals).

The proportion of rotifer metacommunity variation explained by the selected significant variables, considering the environmental and the spatial component together, varied highly among sampling seasons (Fig. 3). In spring, pure environmental effects predominated (64.3%) and the pure spatial effect explained a very low amount of variation (3.0%). On the contrary, the environmental component strongly diminished in autumn (8.4%) in favor of the unexplained component (84.7%), while the pure spatial component slightly increased though it remained low (7.5%). In this sampling period, we did not find effects of the overlap between environmental and spatial components. The selected environmental parameters in the dbRDA were Cl, TP and NH_4_-N in spring, whereas maximum depth of the pond basin was the only environmental parameter selected in autumn. Regarding the spatial variables, the forward selection procedure chose MEMs that represented a combination of both broad- and fine-scale processes in both sampling periods.

### Identifying biogeochemical processes and their controlling factors

**Fig. 4:**
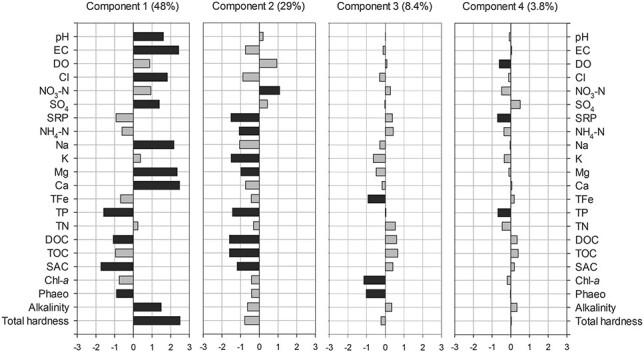
Loadings of the first four Isomap components for physical–chemical parameters. Black bars represent significant correlations (*P* < 0.05) between Isomap components and physical–chemical parameters. The percentage of the total variance in our data set explained by each Isomap component is indicated in parentheses.

The first four Isomap components explained 89.2% of the total variance in our data set. The first two components displayed 48 and 29% of the total variance, while the third and the fourth accounted for 8.4 and 3.8%, respectively. These Isomap components were studied in further detail. The first Isomap component was positively correlated to pH, EC, Cl, SO_4_, Na, Mg, Ca, alkalinity and total hardness (Fig. 4). Non-significant positive correlations were also found for DO and NO_3_-N. The second Isomap component was negatively correlated to nutrients and organic carbon, specifically to SRP, TP, DOC, TOC, SAC, K, NH_4_-N, but also to Na and Mg (Fig. 4). The third Isomap component showed negative correlations with Chl-*a*, Pheo and TFe, while the fourth component was significantly negatively correlated to SRP, TP and DO (Fig. 4).

**Fig. 5:**
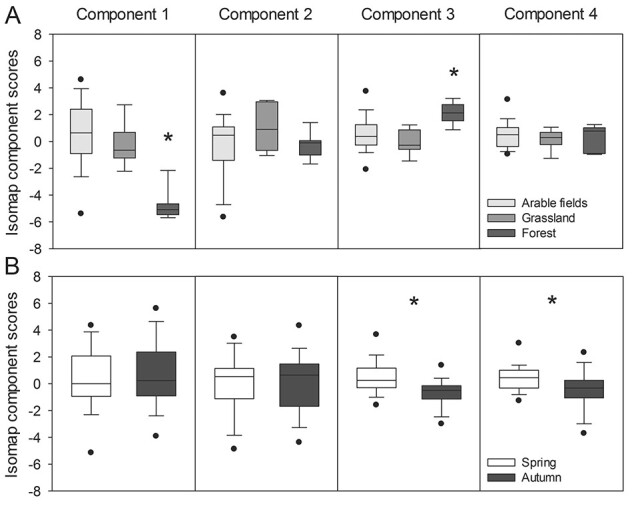
Isomap component scores for (**A**) land use categories and (**B**) seasons. Box plots indicate interquartile ranges (areas within a box), medians (horizontal line within the box), 25th and 75th percentiles (lower and upper box boundaries), and 5th and 95th percentiles (whiskers above and below the box); outliers are shown as solid circles. Asterisks indicate significant differences among land use categories or seasons (*P* < 0.05) based on GLMM results.

The generalized mixed models revealed significant differences in the Isomap components and water physical–chemical parameters with regard to land use (Table SI, Fig. 5A, Fig. S4A and B). The scores of the first and third Isomap component were significantly different among land-use categories. Whereas the former had lower values for forest ponds than for those in grasslands or arable fields, the latter had higher values for forest ponds compared to the other two land use categories (Table SI, Fig. 5A). No significant differences between land use categories were detected for the scores of the second and the fourth components (Table SI, Fig. 5A). We found lower pH, EC, water temperature, Mg, Ca, Na, alkalinity and total hardness values in forest ponds compared to ponds in arable fields and grassland, but higher values for DOC, TOC and SAC (Table SI, Fig. S4A and B). Cl was significantly higher for ponds in arable fields compared to ponds in grassland and forest (Table SI, Fig. S4A). The GLMMs also revealed significant differences in the Isomap components and water physical–chemical parameters with regard to seasonality (Table SI, Fig. 5B, Fig. S5A and B). The scores of the third and fourth Isomap components were significantly lower in autumn than in spring (Table SI, Fig. 5B). However, no significant differences between seasons were detected for the first and second Isomap components (Table SI, Fig. 5B). Significantly, higher values for autumn compared to spring were found for NO_3_-N, K, Mg, Ca, TFe and total hardness (Fig. S5A and B). On the contrary, pH, water temperature, Cl, DOC, TOC, SAC and were significantly lower in autumn (Table SI, Fig. S5A and B). We found significant differences in the first, third and fourth Isomap component with regard to a number of hydrogeomorphic and habitat structure variables (Fig. S6A–H). These variables included hydroperiod, water level, shore width, shore slope and canopy cover. However, no distinct/clear trends along the categories of these variables were detected. Therefore, the effects of these controlling factors on water physical–chemical parameters or rotifer communities were not further explored.

### Effects of biogeochemical processes and their controlling factors on rotifer communities

**Fig. 6:**
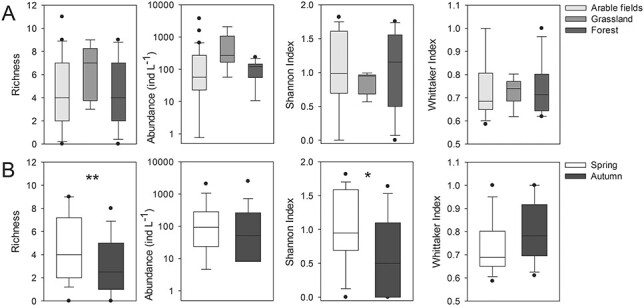
Rotifer abundance and diversity estimates for (**A**) land use categories and (**B**) seasons. Box plots indicate interquartile ranges (areas within a box), medians (horizontal line within the box), 25th and 75th percentiles (lower and upper box boundaries), and 5th and 95th percentiles (whiskers above and below the box); outliers are shown as solid circles. Note that no significant differences (*P* < 0.05) among land use categories or seasons were found based on GLMM results.

The first Isomap component was negatively correlated to rotifer abundance, rotifer richness and the Shannon index, but positively correlated to the Whittaker index (Table II). Conversely, the third component was positively correlated to rotifer richness and the Shannon index, but negatively correlated to the Whittaker index (Table II). No correlations were found between the second or fourth component and the rotifer abundance and diversity estimates. Neither rotifer abundance nor diversity estimates differed significantly among land use categories (Fig. 6A). Both rotifer richness (estimate: −0.53 ± 0.17, df = 26, *t* = −3.15, *P* < 0.01) and the Shannon index (estimate: −0.38 ± 0.14, df = 26, *t* = −2.66, *P* < 0.05) were significantly lower in autumn than in spring (Fig. 6B).

**Table II:** Summary statistics of generalized linear mixed-effects models for the effects of the Isomap components on rotifer abundance, richness, the Shannon index and the Whittaker index in the studied ponds

Response variable	Factor	Estimate	SE	t-value	*P*-value	SD_random_
Rotifer abundance	Intercept	5.48	0.25	21.88	**<0.001**	
	Component 1	-0.33	0.10	-3.22	**<0.01**	
	Component 2	-0.08	0.09	-0.83	0.41	
	Component 3	0.18	0.12	1.50	0.15	
	Component 4	0.04	0.15	0.24	0.81	
	*Latitude*					1.6 × 10^−1^
	*Longitude*					1.6 × 10^−1^
	*Pond name*					1.1 × 10^−4^
Rotifer richness	Intercept	1.37	0.10	13.77	**<0.001**	
	Component 1	-0.11	0.04	-2.64	**0.01**	
	Component 2	0.00	0.04	0.05	0.96	
	Component 3	0.13	0.06	2.15	**0.04**	
	Component 4	-0.04	0.07	-0.54	0.59	
	*Latitude*					6.7 × 10^−1^
	*Longitude*					6.7 × 10^−1^
	*Pond name*					1.3 × 10^−2^
Shannon index	Intercept	0.84	0.08	11.15	**<0.001**	
	Component 1	-0.07	0.03	-2.59	**0.02**	
	Component 2	-0.02	0.03	-0.59	0.56	
	Component 3	0.10	0.05	2.14	**0.04**	
	Component 4	-0.03	0.05	-0.66	0.52	
	*Latitude*					1.6 × 10^−5^
	*Longitude*					1.6 × 10^−5^
	*Pond name*					1.7 × 10^−5^
Whittaker index	Intercept	0.75	0.02	43.26	**<0.001**	
	Component 1	0.02	0.01	2.86	**0.01**	
	Component 2	0.01	0.01	1.17	0.25	
	Component 3	-0.03	0.01	-2.28	**0.03**	
	Component 4	0.00	0.01	0.10	0.92	
	*Latitude*					3.4 × 10^−6^
	*Longitude*					3.4 × 10^−6^
	*Pond name*					3.1 × 10^−2^

Note that forest ponds were excluded from these analyses. Significant *P*-values are shown in bold.

## DISCUSSION

### Environmental variables and rotifer communities

The wide variations found in environmental parameters in the studied ponds reflect high temporal and spatial heterogeneity and confirm the potential of ponds to be hotspots for biogeochemical cycling and biodiversity (Gołdyn *et al*., 2012; Altenfelder *et al*., 2014; Reverey *et al*., 2016). For example, even though all EC values fell within the freshwater range, a 20-fold variation among ponds was observed. Likewise, both TP and TN concentrations fell within the hypertrophic range (Wetzel and Likens, 2000) but showed nonetheless a 20- and 10-fold variation, respectively. Environmental parameters were in the same range and showed similar patterns as ponds from the same area studied by Lischeid *et al*. (2018). Similarly to Lischeid *et al*. (2018), we found high concentrations of alkali (Na, K) and earth alkali ions (Mg, Ca), which those authors attributed to a strong influence of groundwater or geogenic processes.

Our diversity results, with approximately 50% of the genera being represented by a single species and with 30% of the genera recorded in a single pond during the study period, provide evidence that each pond might contribute with unique species to the regional metacommunity and illustrate the high value of individual ponds in the context of aquatic biodiversity conservation. The high potential of small water bodies in agricultural landscapes to enhance local and regional biodiversity and to act as stepping stones between existing aquatic habitats has been described in previous studies, including human-made ponds and wetlands as well as ponds subjected to fish stocking (Williams *et al*., 2004; Céréghino *et al*., 2008; Thiere *et al*., 2009; Kuczyńska-Kippen and Pronin, 2018). Biodiversity should be ideally assessed at the species level (Bozzuto and Blanckenhorn, 2017). Yet, adopting a lower taxonomic resolution such as genus does not necessarily imply a loss of information on the response of plankton to environmental conditions (Machado *et al*., 2015). In the particular case of rotifers at the Schelde estuary, for example, the information on community structure in relation to spatio-temporal variation in environmental factors was comparable at the species and genus level (Azémar *et al*., 2010). Therefore, we consider the insights gained into our study as representative of general biodiversity trends in response to environmental and spatial factors. Furthermore, FlowCAM^®^ technology enables a comprehensive assessment of samples by screening all the individuals present in a sample and yielding corresponding images that can be later retrieved for taxonomic identification.

### Role of environmental versus spatial factors structuring the rotifer metacommunity

Our study revealed distinct seasonal variations in the structure of rotifer communities in ponds, which were mainly driven by fluctuations in the abundance and distribution of the genera *Polyarthra*, *Keratella*, *Lepadella* and *Lecane*. The decrease in rotifer richness and Shannon index observed from spring to autumn indicates a shift from a more diverse and even rotifer assemblage in spring to a less diverse community with markedly dominant species in autumn. This might be explained by the fact that spring, after flooding, is the period of maximum emergence from sediment, when rotifers and other species that diapause in the egg stage are hatching (Hairston *et al*., 2000; Olmo *et al*., 2012).

The structure of the rotifer metacommunity in the ponds seems to be determined by environmental factors acting at the local scale rather than by spatial factors related to regional-scale processes such as dispersal, which is in line with previous studies conducted at comparable spatial scales (Waterkeyn *et al*., 2008; Antón-Pardo *et al*., 2015). The explained variance analysis indicated that niche processes seem to prevail in structuring the rotifer assemblage in spring, with habitat filters selecting different rotifer genera in each pond. Among these filters, and in accordance with our Isomap results, parameters related to EC, internal nutrient recycling and primary productivity were identified by the dbRDA as relevant. In autumn, however, the large unexplained variance indicates that factors other than the abiotic ones we considered in the present study became more relevant. We hypothesize that along the seasonal succession, local biotic interactions, such as competition with macrofilter feeders for food resources or macroinvertebrate predation, shape the rotifer community of each pond specifically, leading to higher divergences. An increase in rotifer community dissimilarly along the seasonal succession in the presence of predation has been observed in an experimental context (Beisner and Peres-Neto, 2009). However, the integration of potential rotifer predators in future studies is needed to confirm this assumption. On the other hand, spatial variables only explained a small fraction of the variance, which points to unlimited dispersal in our study area, probably as a consequence of its relatively small size (220 km^2^) and the frequent presence of well-known zooplankton dispersal vectors such as wind, waterfowl or other animals (Havel and Shurin, 2004; Horváth *et al*. 2014).

### Biogeochemical processes, controlling factors and their effects on rotifer communities

Among the prevailing biogeochemical processes identified by the Isomap analysis, only fertilizer-driven salinization and primary productivity were correlated to total rotifer abundance or diversity. The first Isomap component shows the highest loadings for parameters associated with the freshwater salinization syndrome, i.e. the salinization and alkalinization of freshwater associated to salt pollution by fertilization or fertilizer-induced weathering, among other processes (Kaushal *et al*., 2018). Fertilizers might (i) enhance primary production, leading to increased pH and alkalinity; (ii) contribute bicarbonate and base cations such as Ca, Mg and K and (iii) enhance nitrification and soil weathering, further increasing base cation concentrations (Kaushal *et al*., 2018). The coincident direction of the correlation of this component with DO and NO_3_-N is an indicator for the input of oxidized compounds related to the application of agricultural fertilizers rather than to a possible exfiltration of geogenic compounds into the ponds originating from groundwater. Thus, this component reflects the impact of agriculture via fertilizer input and the resulting freshwater salinization. Lischeid and Kalettka (2012) found similar patterns in ponds in Northeast Germany, but with low explanatory power (11%). They suggested this component as an indicator for anthropogenic pressure on pond water quality, i.e. the impact of agricultural solutes input. The fact that the first component in our study was significantly lower for forest ponds, whereas Cl was significantly higher in arable field ponds, suggests an increasing impact of salinization by fertilizers from forest to arable field ponds. The negative correlation of the first Isomap component with the rotifer abundance, richness and the Shannon index indicates a negative impact of agricultural fertilizers and the associated salinization on the rotifer community. Specifically, the stronger the impact of fertilizers and salinization, the lower is rotifer abundance and the diversity of the rotifer assemblage. EC is known as one of the main factors structuring rotifer assemblages, with rotifer communities being disturbed by increasing salinity (e.g. Bielanska-Grajner and Gladysz, 2010). For instance, higher zooplankton species richness has also been associated with lower EC in temporary Mediterranean ponds and wetlands (Waterkeyn *et al*., 2008; Olmo *et al*., 2016). Similarly to our study, salinity was one of the main drivers of rotifer community structure in a set of shallow water bodies comprising both permanent and temporal sites, with rotifer diversity significantly decreasing with increasing salinity (Malekzadeh-Viayeh and Špoljar, 2012). In addition to EC, Pociecha *et al*. (2015) found particularly calcium and magnesium (which in our study showed very high positive loadings in the first Isomap component) to be the main factors structuring rotifer communities in urban water reservoirs. Furthermore, fertilizer-driven salinization seems to increase rotifer community dissimilarity, as indicated by the positive relationship between the first component and the Whittaker index (beta diversity). Although we did not analyze the turnover and nestedness components of biodiversity in our study (Baselga 2010), we hypothesize that this relationship reflects a positive effect of environmental heterogeneity, and specifically of EC heterogeneity, on species turnover. Heterogeneity in EC might have promoted species turnover between ponds, leading to increased dissimilarity among rotifer communities. The positive correlation between beta diversity and environmental heterogeneity has been shown by a number of studies focused on a variety of taxonomic groups (Harrison *et al*., 1992).

The third Isomap component is representative of primary productivity and thus primary productivity. This component was not significantly correlated to rotifer abundance, which indicates that primary productivity might be mainly controlled by larger zooplankton forms rather than by rotifers (Irfanullah and Moss, 2005). We found a decrease in rotifer richness and the Shannon index with increasing primary productivity. Similar to our study, zooplankton richness decreased with increasing productivity in lakes with watersheds subjected to anthropogenic development (Dodson *et al*., 2000). Those authors proposed competition for edible phytoplankton as well as abiotic factors such as low DO concentrations or high pH in eutrophic water as possible mechanisms explaining this negative relationship. Moreover, the negative correlation of the third component with the Whittaker index reveals that the rotifer community reacts to an increase in primary productivity with increasing dissimilarities in genus composition between the ponds. Chase and Leibold (2002) also found a positive correlation of primary productivity with beta diversity of animals <0.33 mm in ponds. They explained this correlation with the probability of regions with higher productivity for (i) increased environmental heterogeneity, (ii) higher seasonal variation in local species and (iii) multiple stable states, i.e. a different order in which species enter a local community. Furthermore, the dominance of stochastic community assembly processes (ecological drift, dispersal or colonization/extinction dynamics) over deterministic ones (niche filtering) has been proposed to account for the increase in beta diversity with productivity (Chase, 2010). Chase (2010) also found that a higher number of species was able to persist in highly productive experimental freshwater ponds, pointing to low environmental filtering under productive conditions. The significantly higher scores of the third component for forest ponds compared to arable and grassland ponds (Fig. 5A) reflects lower primary productivity in the forest ponds, likely due to light limitation as a result of significantly higher DOC and TOC inputs of terrestrial origin (increased litter input from the surrounding vegetation as well as humic substances from the forest soils) and shading by canopy coverage. The third Isomap component was significantly lower in autumn than in spring, and accordingly, Chl-*a* and Pheo concentrations were significantly higher in autumn.

The second Isomap component reflects internal nutrient recycling processes. The lack of correlation of this component with Chl-*a* suggests that internal recycling mechanisms other than phytoplankton basal metabolism are mainly responsible for nutrient release. Previous findings in one of our studied ponds indicate that nutrients made available via mineralization and phytoplankton basal metabolism were not sufficient to cover phytoplankton P and N requirements, pointing to a limited contribution of phytoplankton to internal nutrient recycling; and a substantial proportion of the bioavailable nutrient concentrations in ponds might originate from submerged macrophyte decomposition and nutrient release from sediments (Onandia *et al*., 2018). It is known that nutrients can influence the abundance and richness of rotifers via trophic cascades (Ji *et al*., 2013). However, no significant correlation of this component with rotifer abundance or rotifer diversity estimates was found. This suggests that internal nutrient recycling via macrophyte decomposition is likely not a relevant process in shaping the rotifer community. The fourth Isomap component can be interpreted as an indicator for redox-dependent P release from the sediments. In agreement with our results, internal P release during hypoxia explained two-thirds of both temporal and spatial variability in physical–chemical parameters in *ca.* 80 ponds in Northeast Germany (Lischeid and Kalettka, 2012). Also, the formation of iron sulfides was suggested to favor P release by reducing the amount of iron available to bind P in one of the studied ponds (Kleeberg *et al*., 2016). There were no significant correlations to rotifer abundance or diversity estimates, showing that the rotifer community in the ponds under study are not significantly affected by sediment nutrient release in a direct way.

Neither rotifer abundance nor richness, the Shannon index or the Whittaker index differed between land use categories. This could be partly due to the long-term impact of intensive agriculture in the region, which has been suggested to result in widespread nutrient loading in shallow groundwater, making ponds connected to the uppermost aquifer highly susceptible to eutrophication (Lischeid *et al*., 2018). In agreement with this hypothesis, TP did not differ between land use categories (Fig. S4A), and all recorded values lay within the hypertrophic range (Wetzel and Likens, 2000). Moreover, *Keratella* species indicative of high trophic state such as *K. cochlearis* Gosse and *K. quadrata* Müller (Cremona *et al*., 2018; Kuczyńska-Kippen, 2020) showed the highest mean abundance during the study period as well as one of the highest frequencies (Fig. 2, Fig. S2).

Note that we only discussed differences related to land use and seasonality, as we did not detect distinct effects of hydrogeomorphic and physical variables, such as hydroperiod or habitat complexity. These factors are known to have potentially strong effects on zooplankton communities (Tavernini 2008; Kuczyńska-Kippen and Joniak, 2016). We attribute the lack of detectable effects in this study to the important role of local environmental factors and unstudied factors (e.g. biotic interactions) in structuring the community, which likely masked the influence of inundation length and aquatic vegetation during our study period. Finally, the biochemical processes identified as prevalent in the ponds, their controlling factors and effects on rotifer communities might be mainly representative of arable fields ponds, as 70% of the studied ponds belonged to this land use category.

## CONCLUSIONS

We found seasonal variations in the predominant processes explaining rotifer metacommunity structure, with the variability explained by spatial factors being consistently low. Nonetheless, further empirical studies addressing the role of biotic interactions, such as interspecific competition of rotifers with mesozooplankton for food resources or predation exerted by macroinvertebrates, are needed to better understand the mechanisms structuring rotifer communities in ponds of natural origin. We identified fertilizer application in the vicinity of the ponds and the associated higher salinity as the predominant process affecting the studied rotifer communities. Fertilizer-driven salinization was related to a decrease in rotifer abundance, richness and the Shannon index, but to an increase in the Whittaker index. The high explanatory power of this process reflects the strong anthropogenic impact on the environmental conditions of the ponds, which was lower in forest ponds than in those surrounded by arable fields or grassland. Primary productivity was also an important factor structuring rotifer communities. Similar to fertilizer-driven salinization, it reduced alpha diversity but increased beta diversity, being also lower in forest ponds than in ponds located in arable fields or grassland. However, neither rotifer abundance nor rotifer diversity estimates differed across land use categories. Widespread eutrophication, caused by long-term intensive agriculture, probably masks any direct effect of agricultural fertilization on the rotifer community in the ponds. Thus, our findings point to an indirect effect of land use on rotifer diversity via fertilizer-driven salinization rather than to a direct land use effect. Finally, around 30% of the rotifer genera were represented by single species and recorded in one single pond, which underlines the high potential of ponds in agricultural landscapes to enhance regional biodiversity, and thus their conservation value.

## Supplementary Material

S1_fbab033

S2_fbab033

S3_fbab033

S4_fbab033

S5_fbab033

S6_fbab033

Table_SI__fbab033
