# Supplementary material for: Key drivers structuring rotifer communities in ponds: insights into an agricultural landscape
Source: J Plankton Res. 2021 May 6;43(3):396–412. doi: 10.1093/plankt/fbab033 (PMC8163045; doi:10.1093/plankt/fbab033)
Supplement: S1_fbab033 [file s1_fbab033.docx]

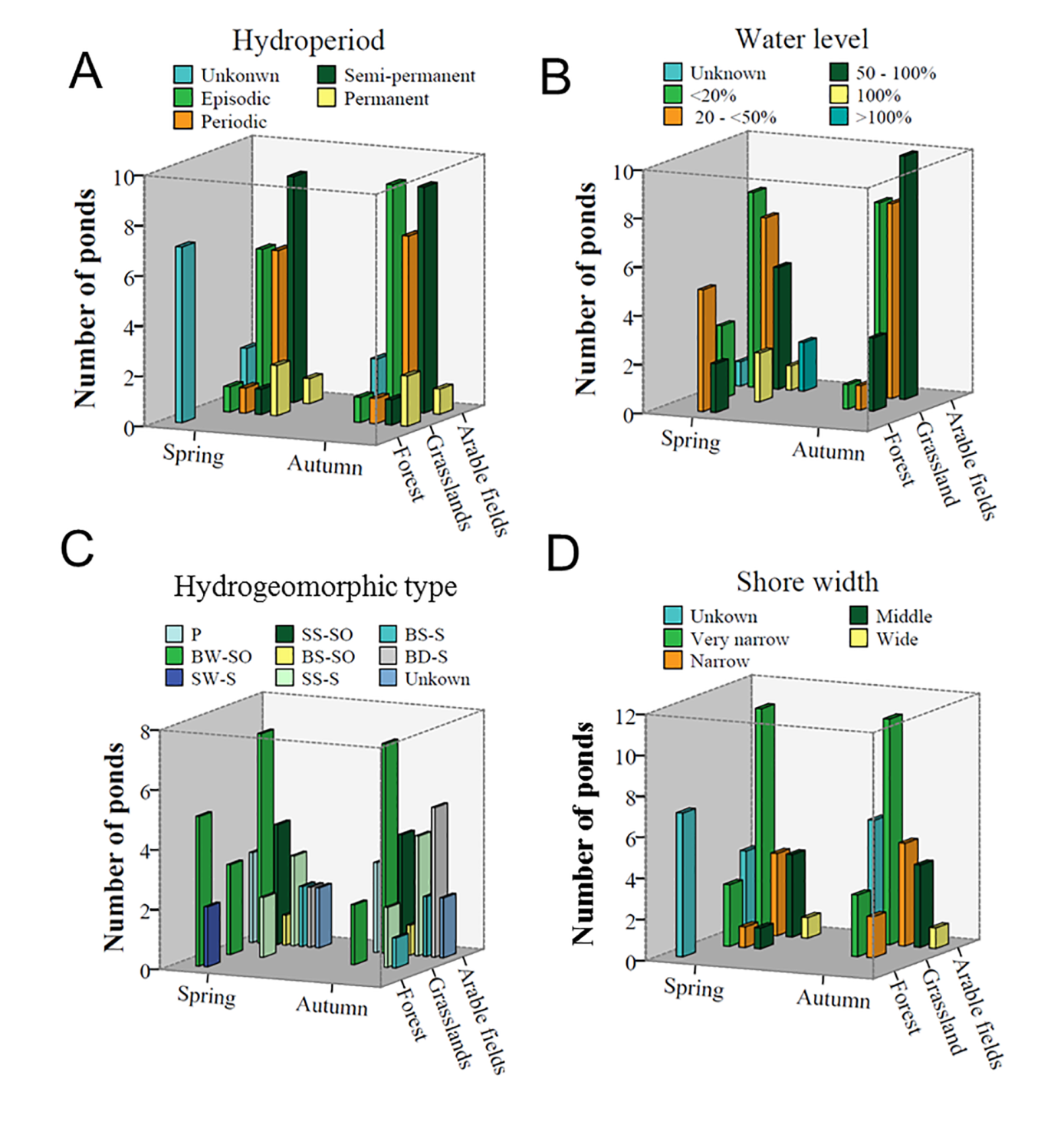


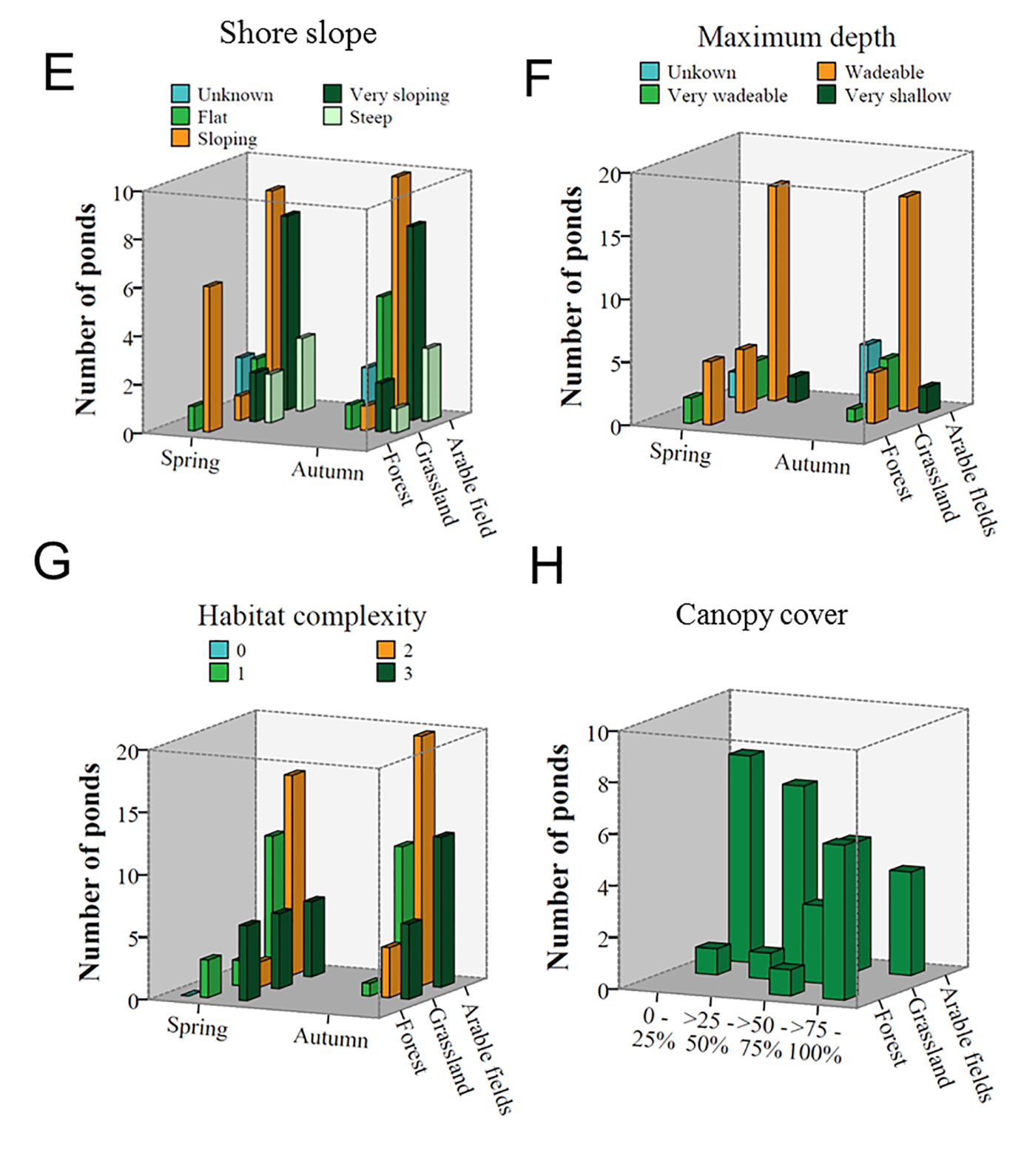


Figure S1. Frequency distribution of ponds belonging to different categories of (A) Hydroperiod; (B) Water level; (C) Hydrogeomorphic type: P, puddle type; BW-SO, big wadeable shore overflow type; SW-S, small wadeable storage type; SS-SO, small shallow shore overflow type; BS-SO, big shallow shore overflow type; SS-S, small shallow storage type; BS-S, big shallow storage type; BD-S, big deep storage type; (D) Shore width: ≤4 m, very narrow; ≤6 m, narrow; ≤10 m, middle; ≤16 m, wide; (E) Shore slope: ≤10%, flat; ≤20%, sloping; ≤30%, very sloping; ≤40%, steep; (F) Maximum depth of the pond basin: ≤1 m, very wadeable; ≤1.6 m, wadeable; ≤2.5 m very shallow; (G) Habitat complexity: number of macrophytes functional groups; and (H) Canopy cover among land use types and seasons. Note that canopy cover was only assessed in spring.
