# Supplementary material for: Key drivers structuring rotifer communities in ponds: insights into an agricultural landscape
Source: J Plankton Res. 2021 May 6;43(3):396–412. doi: 10.1093/plankt/fbab033 (PMC8163045; doi:10.1093/plankt/fbab033)
Supplement: S2_fbab033 [file s2_fbab033.docx]

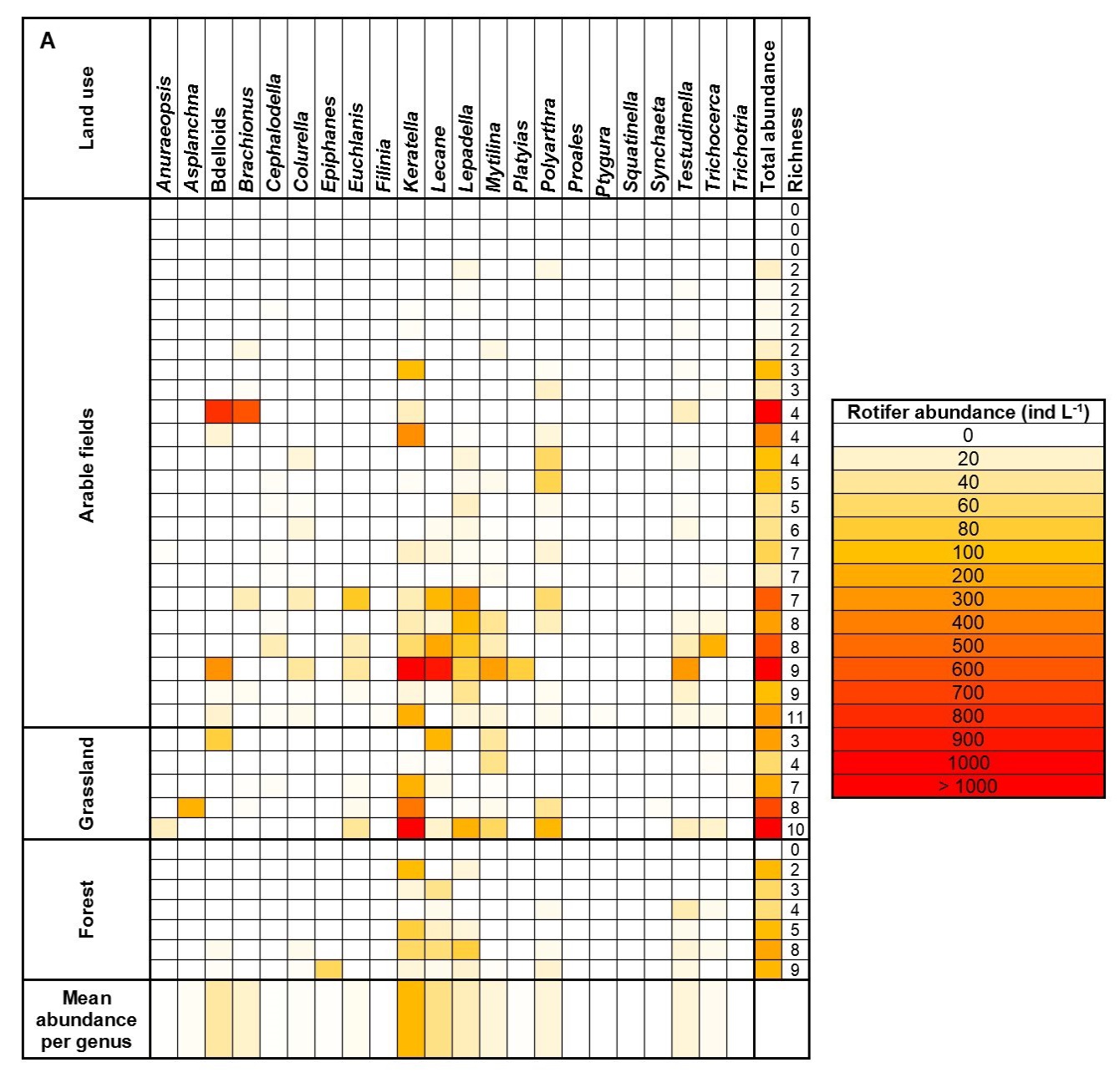


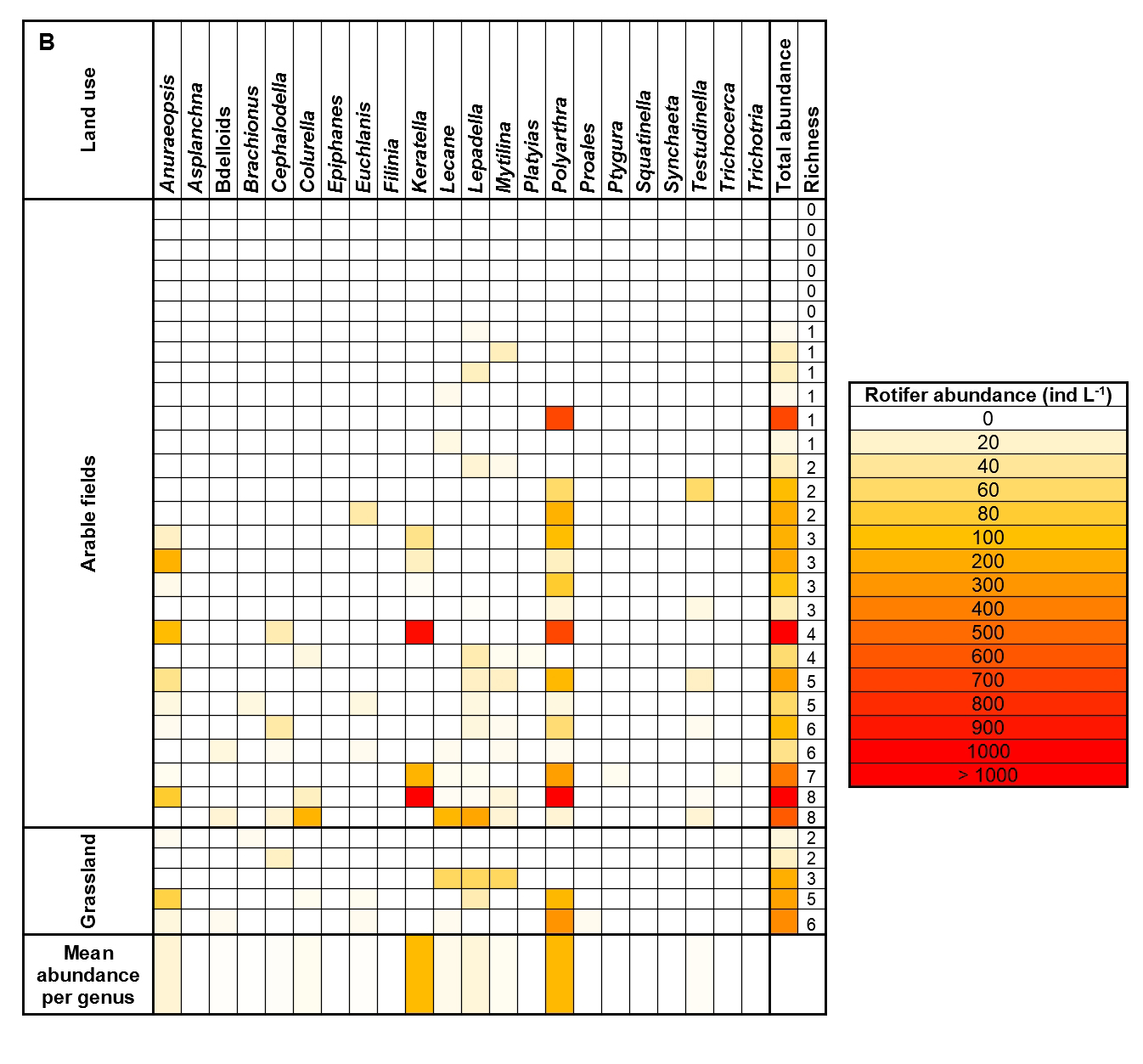


Figure S2. Heatmap of rotifer abundance in (A) spring and (B) autumn in the studied ponds. The data is sorted by increasing richness for each land use category. Individual and mean abundances per rotifer genus and rotifer abundance per pond are presented.
