# Supplementary material for: Key drivers structuring rotifer communities in ponds: insights into an agricultural landscape
Source: J Plankton Res. 2021 May 6;43(3):396–412. doi: 10.1093/plankt/fbab033 (PMC8163045; doi:10.1093/plankt/fbab033)
Supplement: S3_fbab033 [file s3_fbab033.docx]

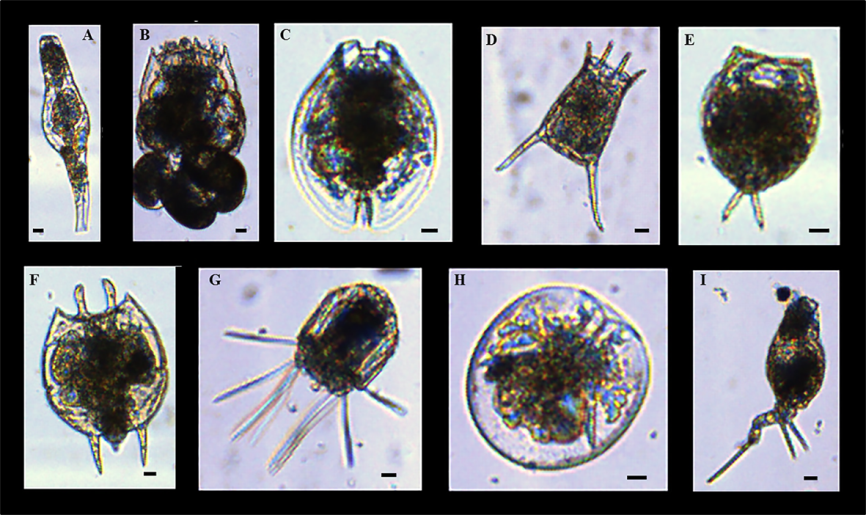


Figure S3. Microphotographs of rotifer taxa captured with an image-based flow cytometer, the FlowCAM® VS, using a 4x objective and a 300 µm flow cell. (A) Bdelloid; (B) *Brachionus*; (C) *Euchlanis*; (D) *Keratella*; (E) *Lecane*; (F) *Platyias*; (G) *Polyarthra*; (H) *Testudinella*; (I) *Trichotria*. The black bar shows a 20 µm scale.
