# Supplementary material for: Key drivers structuring rotifer communities in ponds: insights into an agricultural landscape
Source: J Plankton Res. 2021 May 6;43(3):396–412. doi: 10.1093/plankt/fbab033 (PMC8163045; doi:10.1093/plankt/fbab033)
Supplement: S4_fbab033 [file s4_fbab033.docx]

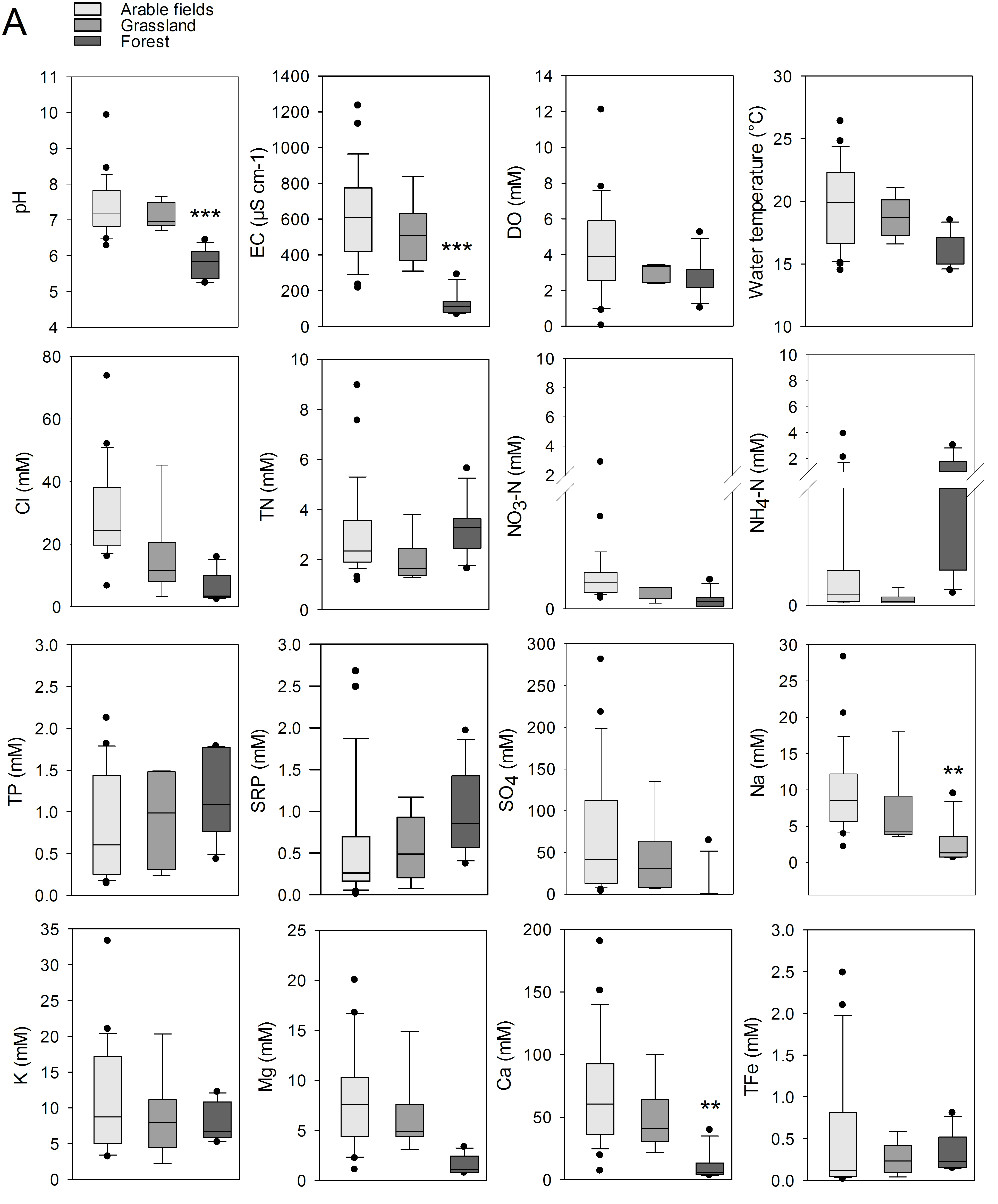


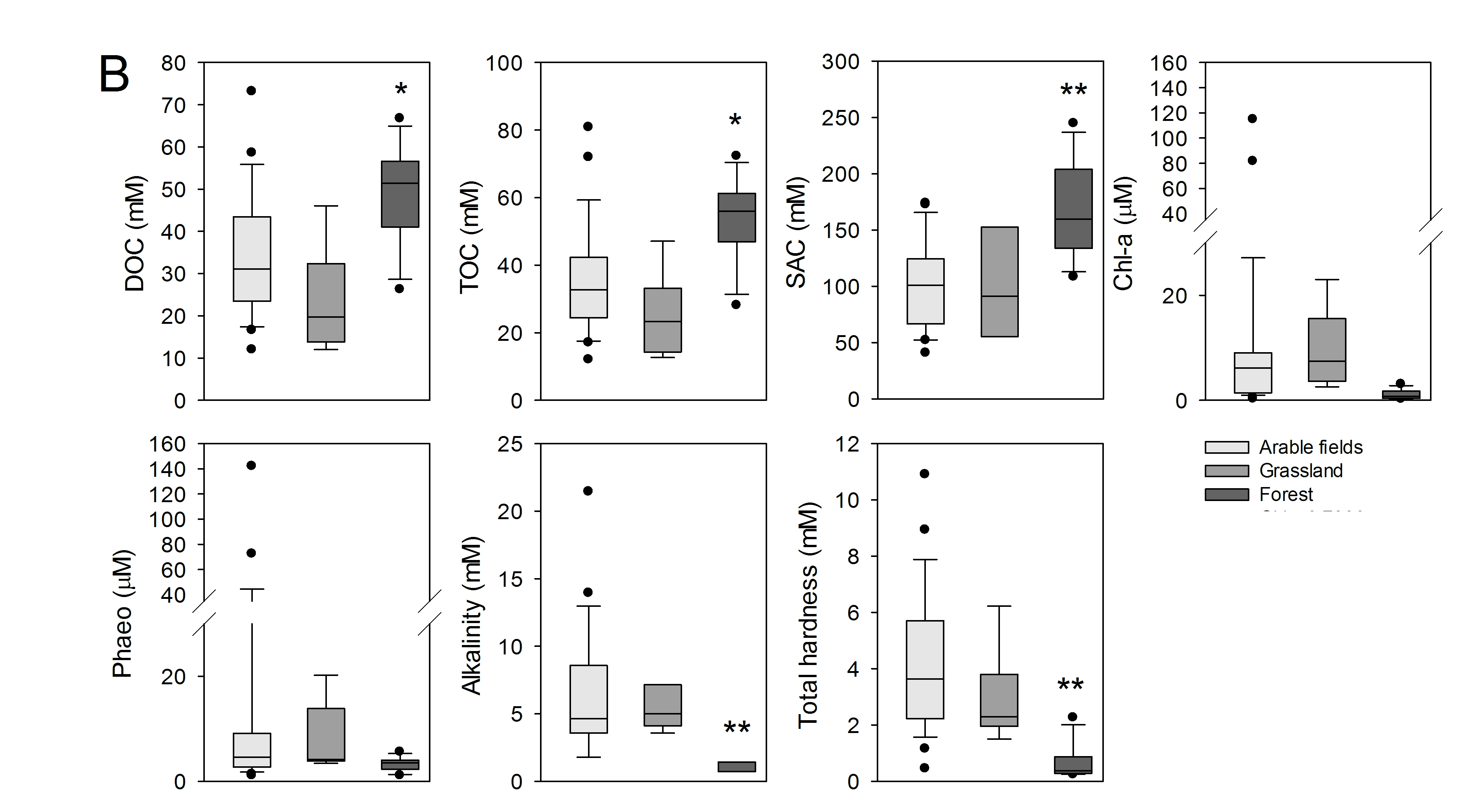


Figure S4A and B. Water physical-chemical parameters of the studied ponds located in different land use categories. Box plots indicate interquartile ranges (areas within a box), medians (horizontal line within the box), 25th and 75th percentiles (lower and upper box boundaries), and 5th and 95th percentiles (whiskers above and below the box); outliers are shown as solid circles. Asterisks indicate significant differences among land use categories (* *P* < 0.05; ** *P* < 0.01; *** *P* < 0.001) based on GLMM results.
