# Supplementary material for: Key drivers structuring rotifer communities in ponds: insights into an agricultural landscape
Source: J Plankton Res. 2021 May 6;43(3):396–412. doi: 10.1093/plankt/fbab033 (PMC8163045; doi:10.1093/plankt/fbab033)
Supplement: Table_SI__fbab033 [file table_si__fbab033.docx]

Table SI. Summary statistics of generalized linear mixed-effects models for significant differences in the Isomap components and water physical-chemical parameters with regard to land use and seasonality.

|  |  |  |  |  |  |  |
| --- | --- | --- | --- | --- | --- | --- |
|  | **Component/Parameter** | **Estimate** | **SE** | **df** | **t-value** | ***P*-value** |
| **Land use** | Component 1 | -5.13 | 1.06 | 33 | -4.81 | <0.001 |
|  | Component 3 | 1.63 | 0.63 | 33 | 2.58 | <0.05 |
|  | pH | -1.57 | 0.31 | 33 | -5.11 | <0.001 |
|  | EC (µS cm^-1^) | -472.55 | 101.89 | 32 | -4.64 | <0.001 |
|  | Water temperature (°C) | -3.96 | 1.21 | 32 | -3.29 | <0.05 |
|  | Cl (mM) | 30.05 | 2.84 | 33 | 10.63 | <0.001 |
|  | Na (mM) | -7.4 | 2..8 | 33 | -3.11 | <0.01 |
|  | Mg (mM) | -6.68 | 1.88 | 33 | -3.55 | <0.01 |
|  | Ca (mM) | -58.41 | 16.77 | 33 | -3.48 | <0.01 |
|  | DOC (mM) | 14.71 | 6.37 | 33 | 2.31 | <0.05 |
|  | TOC (mM) | 18.33 | 6.98 | 32 | 2.62 | <0.05 |
|  | SAC (mM) | 5.48 | 1.68 | 33 | 3.25 | <0.01 |
|  | Total hardness (mM) | -3.47 | 0.97 | 33 | -3.58 | <0.01 |
|  | Alkalinity | -5.48 | 1.67 | 33 | -3.24 | <0.01 |
|  |  |  |  |  |  |  |
| **Season** | Component 3 | -1.15 | 0.36 | 25 | -3.17 | <0.01 |
|  | Component 4 | -0.79 | 0.35 | 25 | -2.22 | <0.05 |
|  | pH | -0.31 | 0.13 | 25 | -2.36 | <0.05 |
|  | Water temperature (°C) | -8.36 | 0.55 | 25 | 15.16 | <0.001 |
|  | Cl (mM) | -5.06 | 2.33 | 25 | 2.17 | <0.05 |
|  | NO_3_-N (mM) | 0.84 | 0.37 | 25 | 2.26 | <0.05 |
|  | K (mM) | 7.28 | 1.8 | 25 | 4.03 | <0.001 |
|  | Mg (mM) | 2.35 | 0.94 | 25 | 2.5 | <0.05 |
|  | Ca (mM) | 17.75 | 7.78 | 25 | 2.28 | <0.05 |
|  | TFe (mM) | 0.66 | 0.2 | 25 | 3.37 | <0.01 |
|  | DOC (mM) | -7.83 | 2.98 | 25 | -2.63 | <0.05 |
|  | TOC (mM) | -7.4 | 3.3 | 25 | -2.22 | <0.05 |
|  | SAC (mM) | -18.12 | 6.98 | 22 | 2.6 | <0.05 |
|  | Total hardness (mM) | 1.08 | 0.5 | 25 | 2.39 | <0.05 |
|  |  |  |  |  |  |  |
|  |  |  |  |  |  |  |
